# Supplementary figures and images for: The gut microbial metabolic capacity of microbiome-humanized vs. wild type rodents reveals a likely dual role of intestinal bacteria in hepato-intestinal schistosomiasis
Source: PLoS Negl Trop Dis. 2022 Oct 24;16(10):e0010878. doi: 10.1371/journal.pntd.0010878 (PMC9633004; doi:10.1371/journal.pntd.0010878)

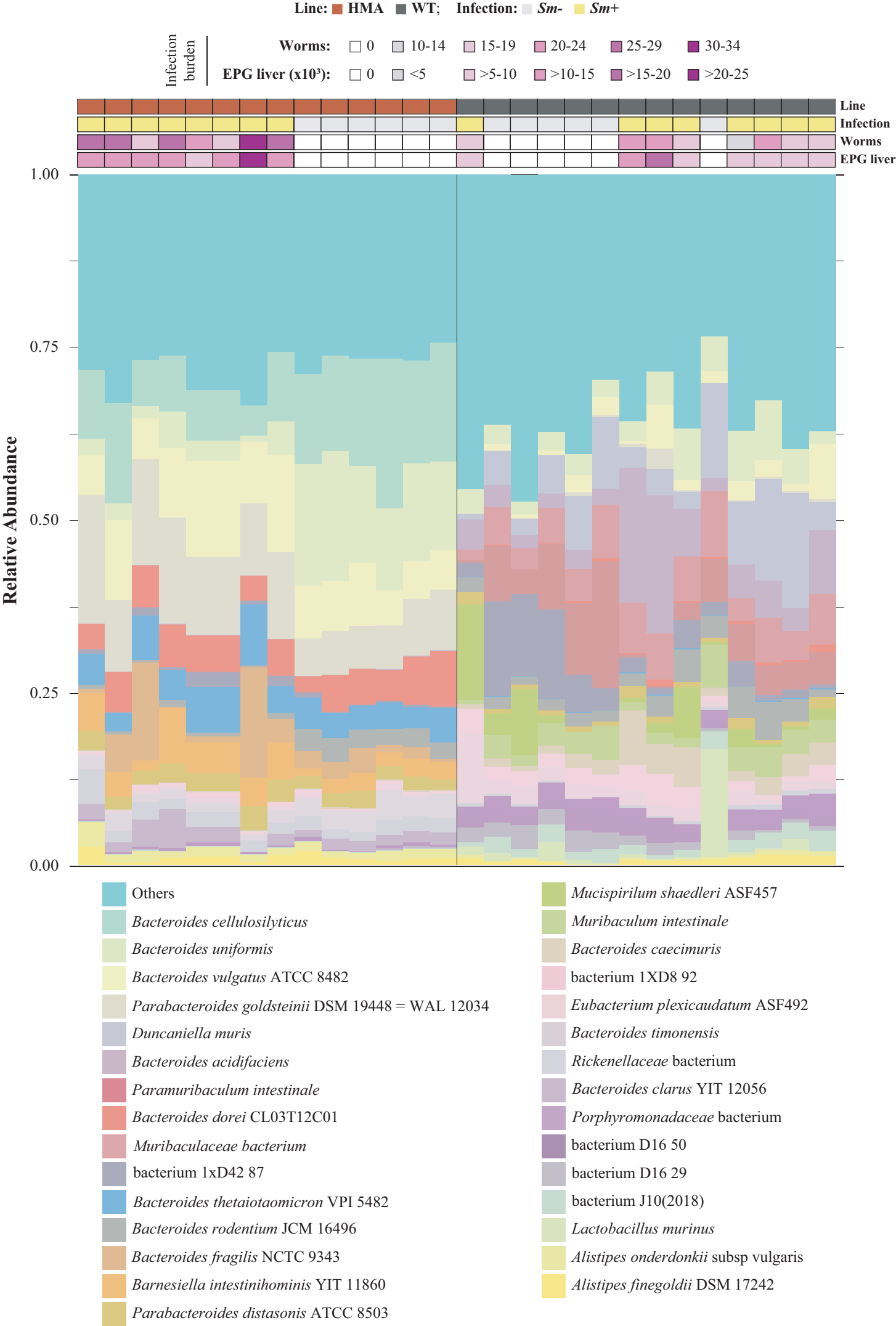

Supplement: S3 Fig — Relative abundances, per sample, of the 30 most abundant bacterial species. Samples are hierarchically clustered according to Pearson correlation between species relative abundances, and explanatory variables are indicated at the top of the figure (i.e., mouse line, infection status and infection burdens; cf. Fig 1. EPG = eggs per gram). (PDF) [file pntd.0010878.s003.pdf]
