# Supplementary material for: The gut microbial metabolic capacity of microbiome-humanized vs. wild type rodents reveals a likely dual role of intestinal bacteria in hepato-intestinal schistosomiasis
Source: PLoS Negl Trop Dis. 2022 Oct 24;16(10):e0010878. doi: 10.1371/journal.pntd.0010878 (PMC9633004; doi:10.1371/journal.pntd.0010878)
Supplement: Attachment — Submitted filename: PNTD-D-22-00561.R1_Rebuttal.docx [file pntd.0010878.s020.docx]

 

6 September 2022

Dear Dr Downs,

**RE: MS. No. PNTD-D-22-00561.R1 (Cortés *et al*.)**

Entitled ‘The gut microbial metabolic capacity of microbiome-humanized vs. wild type rodents reveals a likely dual role of intestinal bacteria in hepato-intestinal schistosomiasis’. *PLoS Neglected Tropical Diseases*. We are very grateful for the reviewers’ and editor’s time and efforts in evaluating this manuscript. The points raised by the reviewers are very constructive and have contributed to enhance the overall value of the manuscript. We have provided detailed responses to individual points in the following rebuttal:

**Reviewer #1**

The authors propose a hypothesis on the role of the intestinal microbiota in the pathogenesis of murine schistosomiasis in wild-type and microbiome-humanized mice, and they test these hypotheses with adequate animal models, adequate infection models and adequate analyses. All methods are clearly described in this or previous works (cited).

Ethical issues are addressed appropriately.

I cannot comment on the statistical analysis reg. sample size but the group sizes are in line with previous publications.

1. The analyses match the proposed hypothesis and the proposed analytical models. The Results are well presented in both Figures and Tables (both of sufficient quality and presented very clearly), and a large body of supplementary data is provided for detailed information if required.

2. The conclusions are fully supported by the data, and the authors fully acknowledge the limitations of their analyses. They also indicate further hypotheses and research questions that should be addressed in future studies. The manuscript leads through the topic from the infection model to analytical details and clearly state that the focus of their conclusions is on the biochemical pathways that differed consistently between infected and uninfected animals in both microbiome model, which complements previous studies focusing more on the bacterial communities themselves.

**RESPONSE**: The authors would like to thank the reviewer for their positive assessment of our manuscript.

3. The manuscript is clear and well presented, and I found only a single error; in the Introduction on page 7: The sentence starts with "Between 3 and 4 weeks" and "(*S. mansoni* and *S. mansoni*)" I believe should be changed to "(*S. mansoni* and *S. japonicum*).

**RESPONSE**: Thank you for spotting this inaccuracy, which has now been addressed.

4. In the cited literature some articles (Frontiers series) are written in capital letters, this does not seem to be the citation style of the journal, but this will likely be changed during the final editing.

**RESPONSE**: Agreed and addressed.

5. This study describes changes in the microbiota upon *Schistosoma mansoni* infection in two different mouse models, a wild type and a humanized microbiome model. The results indicate that not only bacterial communities, but the microbial metabolome change with infection irrespective of the mouse strain (ruling out a possible influence of the host). The possible meanings of these findings are well discussed in relation to previous works on the topic of the role of the gut microbiome in infections with helminths, and help to form a more complete picture on the complex pathogenesis of chronic helminth infections and the interactions not only between host and parasite but involving the third party in this, the microbiome. The study is well designed, well presented and adds significant novel data to this complex topic.

Should shortening be required, the biology of *Schistosoma* (all three major species) included in the introduction probably does not need so much detail, however, the information is not completely redundant as pathogenesis is also the topic of the discussion.

**RESPONSE**: The authors would like to thank the reviewer for their overwhelmingly positive comments.

**Reviewer #2**

This study from Cortes et al examines schistosome-induced changes to gut microbiome both in WT C57BL/6 mice and C57BL/6 reconstituted with a human microbiota. This builds on previous work from these groups showing *Schistosoma mansoni* infection alters microbial composition in Swiss-Webster mice (Jenkins Sci Rep 2018) and the two C57BL/6 models used in the current study (Cortes Front Immunol 2020, presumably using the same samples). The key difference this study uses whole genome sequencing rather than 16S rRNA analysis so giving species level resolution here. Whilst many of the headline findings have been reported in the previous studies (i.e., schistosome infection alters microbiota; this occurs in both WT and HMA mice; these two groups are very different), species level analysis now gives us a more in-depth picture of infection-induced changes and also allows a more extensive discussion of the implications (for instance, metabolic pathway analysis and potential effects on host immunity). Much of this is necessarily speculative but at the very least provides a set of testable hypotheses for future work.

**RESPONSE:** The authors would like to thank the reviewer for their positive assessment of our work.

Minor comments:

1. At times the analysis does feel a little overlong. For instance, the plasmid/horizontal gene transfer section is perhaps too speculative.

**RESPONSE**: Many thanks for this constructive comment. While Reviewer 3 raises the opposite suggestion, i.e., conducting additional analyses to measure HGT rates and/or investigating putative mechanisms of resistance (see comment no. 4, reviewer 3), we however believe that such analyses would be beyond the scope of the current manuscript and contribute to further lengthen the text. We therefore believe that a just compromise would be to maintain this section as is, and highlight the need for more in-depth investigations on these aspects (as suggested by reviewer 3) in the Discussion section.

2. Fig. 2C well shows clear separation between naïve and infected HMA mice (not the case for WT animals). It is not easy from Table S3/S4 to identify the pathways that drive this separation (whilst individual species are in Fig. 3).

**RESPONSE:** Many thanks for this comment. Significant differences in the relative abundances of selected pathways were detected between *Sm+* and *Sm-* mice of each WT and HMA by both MaAsLin2 and LEfSe approaches. A large number of differentially abundant pathways were identified between *Sm+* and *Sm-* mice of each line; these are listed in Supplementary Tables S5.R1 and S6.R1. Within each table (sheet MaAsLin2&LEfSe), each differentially abundant pathway is linked to the corresponding infection group (i.e., *Sm*+ or *Sm*-) by means of colors (see column “Associated group” next to the “Pathway” column).

3. Similarly, a table showing the metabolic pathways that change in both WT and HMA (page 14) would be useful, highlighting the 3 that are consistent (and the 8 that are not). As it stands, TRPSYN-PWY is the standout discussed in depth.

**RESPONSE:** Many thanks for this comment. The information requested by the reviewer has now been made available from Supplementary Table S7.R1.

4. The 2020 Frontiers paper correlated changes in microbiota with parasite burden. Is it possible to do the same here? Assuming these are the same animals, there is a reasonable spread of worms (and eggs) within groups that may be informative, especially where hierarchical clustering (Fig. 1) groups some infected WT mice with naives – are these the ones with lower worm burdens? I expect this is too simplistic given the nature of the outliers, but would be good to see.

**RESPONSE:** Agreed and addressed.

**Reviewer #3**

This study by Cortés et al presents an in-depth investigation of microbial traits associated with *S. mansoni* infection in two mice strains – a wild type strain and a strain with a humanized microbiota. They used shotgun sequencing to compare species-level taxonomic as well as functional profiles between controls and rodents infected with *S. mansoni*. The study provides new and original results and discussion points. However, the manuscript could potentially be improved by a few additional considerations described below:

Major:

1. Decrease in gut bacterial richness in HMA at day 50 – I understand that it might be difficult (or impossible) but comparing the original donor sample to the engrafted community could also be of interest. Was the decrease in diversity also associated with a decreased in bacterial load?

**RESPONSE:** The authors thank the reviewer for this pertinent comment and agree that comparing engrafted bacterial community in HMA mice with the original donor sample would have been of great interest. However, for reasons beyond our direct control (the line of HMA mice used in this study was generated for purposes unrelated to our project), no sequencing data is available for the original donor sample. We also regret that we are unable to quantify 16S gene content in our samples as whole amounts of DNA extracts were subjected to shotgun metagenomics sequencing.

2. An additional figure (or supplementary figure) providing a visual comparison of the taxonomic composition for each individual mouse (e.g., bar charts) sorted according to their position in the heatmap cluster would complement the heatmap well.

**RESPONSE:** Agreed and addressed; please refer to Supplementary Figure S3.R1.

3. Does the enrichment of individual SCFA production/metabolism-related pathways also reflect an enrichment at a higher metacyc functional level (e.g., Fermentation to Butanoate)? If yes, a comparison of the *Sm*-/*Sm*+ abundance ratios between mice strains could be interesting.

**RESPONSE:** Many thanks for this interesting comment. While MetaCyc pathways are classified into hierarchical classifications, they are not directly hierarchical with each other (like, for instance, GO terms) and, therefore, abundance at higher MetaCyc functional levels cannot be calculated from our data. There is a lot of complexity that is summarized by HUMAnN3 at the pathway level, as each pathway maps to many reactions, which, in turn, map to many genes across many species. The reactions comprising each pathway could be intersected to see how related they are in terms of sharing reactions; however, we feel like it would be too distracting and confusing to go into detail about the relatedness of pathways throughout the manuscript.

4. The discussion about HGT/AMR is interesting. However, since the study was conducted using shotgun sequencing, more in-depth analyses could be performed using dedicated software to measure e.g., HGT rates (with metachip or daisysuite) or to investigate mechanisms of resistance more in detail (e.g., if plasmid driven or not?).

**RESPONSE:** The authors would like to thank the reviewer for this interesting suggestion and agree completely; however, we feel that such in-depth analyses are beyond the scope of the current manuscript, and would moreover further expand the (already substantial) breadth of information provided. Reviewer 2 suggested to reduce this section (see comment no. 1, reviewer 2). Therefore, we believe that a just compromise would be to leave this section as is, whilst addressing the need for further investigations in this area in the Concluding Remarks section.

Minor:

5. Missing information about the sequencing results (e.g. sequencing depths etc..)

**RESPONSE:** Addressed.

6. “(*S. mansoni* and *S. mansoni*)”

**RESPONSE:** Many thanks for spotting this inaccuracy, which has now been addressed.

7. Section results/discussion could be separated for improved clarity.

**RESPONSE:** The authors would like to thank the reviewer for this suggestion. However, given the complexity of the experimental design, which includes taxonomical and functional comparisons between (i) mouse lines and (ii) infected and naïve animals within each line, we strongly believe that discussing each set of findings individually is helpful to readers.

8. A visual representation accompanying the statements made about the differences in amino acid metabolism (end of page 14) would be useful.

**RESPONSE:** Agreed and addressed; please refer to Supplementary Figure S7.R1.

9. “Expansion of this pathway in WT *Sm*+ mice was linked to two Lachnospiraceae bacteria (i.e., A4 and 10 1) as well as to *Bacteroides vulgatus*; nonetheless, the individual contribution of these bacteria to the overrepresentation of the pathway was not statistically significant (Fig 4B). In HMA, in contrast, expansion of TRPSYN-PWY following infection was significantly linked to *B. vulgatus*, together with other low abundant bacteria (Fig 4B).” Would it be possible to compare the actual genes *B. vulgatus* contributed to the pathways?

**RESPONSE:** The HUMAnN3 pipeline that we utilized does not export this data by default, and it is a complicated output since each pathway maps to many reactions, and each reaction can contain many genes. Each gene can then be assigned to many species, and reactions overlap between pathways, and genes overlap between reactions. All of these associations are contained in complex database association files and intermediate output files which are not easily accessible and cannot be easily shared. Therefore, exporting gene data for every pathway in a supplementary table is extremely challenging, and the output would be many millions of rows of data with multiple associations.

Nonetheless, we have parsed out the genes for the TRPSYN-PWY specifically (accessible via the following link: <https://links.uv.es/Blgm0aY>). The linked table shows that, out of all genes in the L-tryptophan biosynthesis pathway (TRPSYN-PWY), the two most highly expressed across samples in the HMA gut microbiome were from *B. vulgatus*. These were (i) Anthranilate phosphoribosyltransferase, which belongs to the PRTRANS-RXN reaction, was 1.59-fold higher in infected HMA compared to uninfected (P = 0.038 by two-tailed T-test with unequal variance), and had an average abundance of 1417.4 reads per kilobase (RPK), compared to an average of 72.9 across TRPSYN-PWY from other species, and (ii) N-(5'-phosphoribosyl)anthranilate isomerase, which belongs to the PRAISOM-RXN reaction (1.44-fold higher in infected, P = 0.102, 1172.9 RPK). In contrast, the two most abundant genes for the TRPSYN-PWY in the WT samples were (i) Anthranilate phosphoribosyltransferase (PRTRANS-RXN reaction) from both *Lachnospiraceae* bacterium A4 and an unclassified bacterium, and (ii) Indole-3-glycerol phosphate synthase (IGPSYN-RXN reaction) again from *Lachnospiraceae* bacterium A4. In terms of *B. vulgatus* gens contributing to the TRPSYN-PWT in the WT, Anthranilate phosphoribosyltransferase was 1.76 higher in infected mice (P = 0.402, 153.7 RPK), whereas N-(5'-phosphoribosyl)anthranilate isomerase was 1.72-fold higher (P = 0.724, 9.7 RPK).

We thank the reviewer for this very pertinent suggestion; however, in our opinion, the results of the analysis described above would not add significant new information to the manuscript; we have therefore opted to exclude these extra-analyses from our manuscript in order to keep the focus of the readers on main findings.

In conclusion, we consider that we have rigorously addressed each of the points raised by the reviewers and revised the text accordingly. Clearly, the suggestions, comments and criticisms have led to an enhanced version of the manuscript, which we now consider to meet the standard for publication in *PLoS Neglected Tropical Diseases*.

We look forward to your final deliberation.

Yours sincerely,

Cinzia Cantacessi

*On behalf of all authors*.
